# NAP1L1: A Novel Human Colorectal Cancer Biomarker Derived From Animal Models of *Apc* Inactivation

**DOI:** 10.3389/fonc.2020.01565

**Published:** 2020-08-11

**Authors:** Cleberson J. S. Queiroz, Fei Song, Karen R. Reed, Nadeem Al-Khafaji, Alan R. Clarke, Dale Vimalachandran, Fabio Miyajima, D. Mark Pritchard, John R. Jenkins

**Affiliations:** ^1^Institute of Systems, Molecular and Integrative Biology, Henry Wellcome Laboratory, University of Liverpool, Liverpool, United Kingdom; ^2^Faculty of Medicine, Federal University of Mato Grosso (UFMT), Cuiaba, Brazil; ^3^INFRAFRONTIER GmbH, Neuherberg, Germany; ^4^Wales Gene Park, Division of Cancer and Genetics, Cardiff University School of Medicine, Cardiff, United Kingdom; ^5^European Cancer Stem Cell Research Institute, Cardiff University School of Biosciences, Cardiff, United Kingdom; ^6^Department of Colorectal Surgery, Countess of Chester Hospital NHS Foundation Trust, Chester, United Kingdom; ^7^Molecular Epidemiology Laboratory, Oswaldo Cruz Foundation, Eusebio, Brazil

**Keywords:** colorectal cancer, biomarkers, *Apc*, NAP1L1, prognosis, survival

## Abstract

**Introduction:**

Colorectal cancer (CRC) is the second leading cause of cancer death worldwide and most deaths result from metastases. We have analyzed animal models in which *Apc*, a gene that is frequently mutated during the early stages of colorectal carcinogenesis, was inactivated and human samples to try to identify novel potential biomarkers for CRC.

**Materials and Methods:**

We initially compared the proteomic and transcriptomic profiles of the small intestinal epithelium of transgenic mice in which *Apc* and/or *Myc* had been inactivated. We then studied the mRNA and immunohistochemical expression of one protein that we identified to show altered expression following *Apc* inactivation, nucleosome assembly protein 1–like 1 (NAP1L1) in human CRC samples and performed a prognostic correlation between biomarker expression and survival in CRC patients.

**Results:**

*Nap1l1* mRNA expression was increased in mouse small intestine following *Apc* deletion in a *Myc* dependant manner and was also increased in human CRC samples. Immunohistochemical NAP1L1 expression was decreased in human CRC samples relative to matched adjacent normal colonic tissue. In a separate cohort of 75 CRC patients, we found a strong correlation between NAP1L1 nuclear expression and overall survival in those patients who had stage III and IV cancers.

**Conclusion:**

*NAP1L1* expression is increased in the mouse small intestine following *Apc* inactivation and its expression is also altered in human CRC. Immunohistochemical NAP1L1 nuclear expression correlated with overall survival in a cohort of CRC patients. Further studies are now required to clarify the role of this protein in CRC.

## Introduction

Colorectal cancer (CRC) is the third most common cancer type and the second leading cause of cancer death worldwide (1). Deaths usually result from recurrent and metastatic disease. Most international guidelines recommend chemotherapy to reduce the risk of recurrence in stage III tumors and to prolong survival in stage IV cancers (2). Conversely, chemotherapy is generally not used in stage I and most stage II tumors. However, some patients with low-risk stage III disease may respond well following courses of chemotherapy that are shorter than the 6-month standard schedule, although the definition of “low-risk” has not been well established in this scenario (3, 4). Additionally, almost 20% of patients who have stage II tumors and who are therefore considered to have low-risk disease, relapse and eventually die from cancer progression (5). There is currently no accurate biomarker to better define prognosis within stage groups. Therefore, biomarkers for prognostic stratification in CRC have the potential of improving the treatment decision-making process (4, 6). We hypothesized that studying molecular mechanisms that are known to be involved in CRC development might yield promising novel biomarkers for this disease.

*Adenomatous polyposis coli* (*APC*) inactivating mutations are the earliest and most common genetic alterations in the adenoma-carcinoma sequence that leads to CRC (7). Such mutations result in the accumulation of β-catenin within cells and activation of the Wnt signaling pathway (8). Animal models of *Apc* inactivation demonstrate epithelial transformation and tumor formation mirroring cancer development (9–11). One of these models is the *AhCre^+^Apc^fl/fl^* mouse, an animal bearing loxp-flanked *Apc* alleles and a *Cre*-recombinase transgene. When this animal is exposed to β-naphthoflavone, Cre-recombinase transcription is activated resulting in the deletion of loxp-flanked *Apc* alleles specifically in the small intestinal epithelium, thus causing an acute activation of the Wnt pathway (9). The *Apc*^Min/+^ mouse has a germline mutation in one *Apc* allele simulating a familial adenomatous polyposis (FAP) patient, and spontaneously develops multiple intestinal neoplasms during its life-span (12, 13). The *Myc* gene is a Wnt-target that plays an essential role in the development of malignant phenotypes upon *Apc* inactivation (14, 15). We hypothesized that the analysis of mouse models of *Apc* and *Apc/Myc* deletion could lead to the discovery of genes or proteins with potential clinical use as human CRC biomarkers.

Our group has previously published a proteomic evaluation of an animal model of CRC based on acute *Apc* deletion (*AhCre^+^Apc^fl/fl^* mouse) (16). A 4-plex iTRAQ labeling analysis identified 126 proteins that were significantly altered upon *Apc* deletion (76 up- and 50 down-regulated) and which were proposed as Wnt targets. We have now performed an additional analysis of this dataset by comparing the protein list with the transcriptomic data generated using Affymetrix arrays and intestinal tissues from the same mice (14). This study used *Apc*–deficient (*AhCre^+^Apc^fl/fl^Myc^+/+^)* and double-mutant *Apc*-*Myc* deficient (*AhCre^+^Apc^fl/fl^Myc^fl/fl^*) mice to identify *Myc* dependant Wnt pathway genes following *Apc* inactivation. We investigated whether there were any genes/proteins that showed congruent findings in both analyses according to strict criteria. One protein, nucleosome assembly protein 1 like 1 (NAP1L1) was identified that met our criteria.

We therefore analyzed the expression of *Nap1l1* in *Apc*–deficient (*AhCre^+^Apc^fl/fl^Myc^+/+^*) and double-mutant *Apc*-*Myc* deficient (*AhCre^+^Apc^fl/fl^Myc^fl/fl^*) mice to assess whether its expression was *Myc*-dependant and therefore a potential biomarker of Wnt activation. Following confirmation of our findings, we investigated the mRNA and protein expression of NAP1L1 in tumor and adjacent normal mucosa samples from patients with CRC as well as colonic tissues from unaffected individuals.

## Materials and Methods

### Animal Samples

Mouse experiments were performed with UK Home Office approval with personal and project licenses (30/2737 and 30/3279) and according to ARRIVE guidelines and following local ethical review by the Cardiff University Animal Welfare Ethical Review Panel. The transgenic mice that were used in this study were generated and maintained at the University of Cardiff as previously described in (14). Small intestinal epithelial cell extracts were generated from these mice following injection of beta-naphthlaflavone as described by Hammoudi et al. (16).

### qPCR RT-PCR

For the mouse small intestinal tissue samples and human CRC samples obtained in the United Kingdom (Wales cohorts 1 and 2), total RNA was used to synthesize first strand cDNA using a VersoTM cDNA Kit (Thermo Scientific) and anchored oligo-dT primers (Thermo Scientific) according to the manufacturer’s instructions. Single-stranded cDNA samples were amplified in a Polymerase chain reaction (PCR) using sequence-specific primers (Eurogentec) and probes from the Universal Probe Library (Roche) that were designed using the Universal ProbeLibrary Assay Design Centre, using PCR Master mix (Roche) and a light cycler 480 (Roche) (see Supplementary Table 2 for primers and probes used).

For the human CRC samples recruited in Brazil, RNA was extracted using the RNeasy^®^ Mini Kit (Qiagen, Hilden, Germany). cDNA was produced using TaqMan^®^ Reverse Transcription Reagents kit (Applied Biosystems, Carlsbad, CA, United States) according to the manufacturer’s protocol. Quantitative PCR reactions were performed using the 7500 Fast Real-Time PCR System (Applied Biosystems, Foster City, CA, United States) (see Supplementary Table 3 for expression assay specifications).

### Proteomic and Microarray Comparison Analysis

We set out to determine the subset of genes/proteins, which demonstrated upregulation of both protein and mRNA following *Apc* deletion, but no increase in expression in *AhCre^+^Apc^fl/fl^Myc^fl/fl^* mice. The MAXD/View-Program data files from the microarray analyses were used to calculate gene expression fold changes in the intestinal tissues from the various transgenic mice. These data were then combined with our previously published proteomic profile data (14) in which we identified proteins which showed a greater than 1.2 fold increase in expression following *Apc* deletion, using iTRAQ analysis.

The following features from the DNA microarray analysis were used to identify candidate *Myc* dependant Wnt pathway proteins: (i) a statistically significant (*p* < 0.05) greater than 2 fold increase in expression in *Apc*–deficient (*AhCre^+^Apc^fl/fl^*) mice compared to wild-type mice (APC:WT); (ii) no significant increase in expression in the double-mutant *Apc-Myc* deficient (*AhCre* + *Apc^fl/fl^ Myc^fl/fl^*) mice when compared to the wild-type mice (a ratio value of 1:1, with boundaries of 0.75 and 1.25) (APCMYC:WT) and (iii) a *AhCre^+^ Apc^fl/fl^ Myc^fl/fl^* to *AhCre^+^Apc^fl/fl^*, ratio < 0.5 with *p* < 0.05 (APCMYC:APC) and findings being confirmed by at least three Affymetrix probes corresponding to the protein.

### Human Samples and Ethics

#### United Kingdom Cohorts

Total RNA samples from CRC tumor tissues and adjacent uninvolved colonic mucosa (18 patients) were obtained from the Wales Cancer Bank (Wales cohort 1) with the ethical approval and informed consent that is associated with this tissue bank, and these samples were used in the initial gene expression studies. There were 9 male and 9 female patients, with 6 samples from stage I, 6 from stage II and 6 from stage III CRC and mean patient age was 69.3 years. Another set of 30 matched sample pairs was subsequently obtained from the same Tissue Bank (Wales cohort 2) and these were analyzed separately for validation of the findings. Wales cohort 2 had 9 samples with stage I, 8 samples with stage II and 13 samples with stage III CRC and the mean patient age was 69.4 years.

A tissue microarray (TMA) was constructed using samples from 19 CRC patients recruited at the Countess of Chester Hospital NHS Foundation Trust (Chester, United Kingdom) again with informed consent and local ethics committee approval (12/NW/0011). Cancer tissues were available for all cases (5 cases with stage I, 3 cases with stage II, 5 cases with stage III and 6 cases with stage IV CRC), whilst normal adjacent mucosa was only obtained for 8 of them. The analysis of this cohort was part of the immunohistochemistry (IHC) validation study and the findings are presented in Supplementary Figure 2.

#### Brazil Cohort

Fresh frozen tissues from tumors removed from 25 CRC patients and normal colonic tissues from 10 normal individuals (who had a normal colonoscopy on a bowel cancer screening program) were collected, after informed consent was obtained, and were analyzed in the gene expression studies. The CRC samples were from 17 males and 8 females with stage I: 7; II: 3; III: 8 and IV: 7 CRC and the mean patient age was 55.9 years. The normal samples were from 3 males and 7 females and the patients had a mean age of 54.7 years.

For the initial Brazilian IHC study, samples from 32 patients were prospectively collected in Cuiaba – Brazil between January 2013 and August 2015. Informed consent was obtained. Fragments from the tumor and from the normal adjacent mucosa (at least 10 cm from the tumor) were fixed in 10% buffered formalin for 24 h, and then processed into paraffin blocks. Patient characteristics are described in the results section below.

For the IHC prognostic study, samples were obtained for 75 CRC patients from the archives of two pathology laboratories in Cuiaba, Brazil (Laboratorio São Nicolau and the Julio Muller University Hospital Pathology Lab). Inclusion criteria were: (i) 4 or more years since diagnosis, (ii) presence of tumor tissue in the paraffin block, (iii) traceable patient survival information, and (iv) survival for at least 30 days after surgery. We tracked the patient’s current health service to obtain mortality information. Alternatively, if no clinical information was available, we checked the Brazilian electronic death database “*Sistema de Informacao de Mortalidade*” which records all deaths and their causes. Overall survival was recorded as the interval between diagnosis and death from any cause (when death had occurred) or the date when the database was last checked (when death had not occurred).

This research was approved by the Committee for Research Ethics of the Hospital Universitario Julio Muller – Federal University of Mato Grosso, Cuiaba – Brazil, and by the Brazilian National Commission for Research Ethics (CONEP), decision number: 1.628.901.

### Immunohistochemistry in the Validation Study

Four μm sections from paraffin blocks were subjected to standard protocols for IHC. Tris-buffered saline-tween 0.1% (TBS-T) was used for all washes. In summary the main steps were: dewaxing in xylene twice; endogenous peroxidases block using 3% hydrogen peroxide in methanol; rehydration in decreasing concentrations of ethanol solutions and, finally, distilled water; heat-induced epitope retrieval using 10 mM citrate buffer pH 6.0 in a microwave oven at 800 W for 20 min; block using 10% normal goat serum (Dako, Ely, United Kingdom) in TBS-T for 45 min at room temperature; primary rabbit anti-NAP1L1 antibody (cat. number ab33076, Abcam, Cambridge, United Kingdom) 1:4,000 in 10% normal goat serum in TBS-T overnight at 4°C in a humid chamber; biotinylated secondary goat anti-rabbit antibody (Dako, Ely, United Kingdom) solution 1:200 in 10% normal goat serum for 30 min at room temperature; avidin-biotin-peroxidase complex (Vectastain Elite ABC kit – Peterborough, United Kingdom) for 30 min at room temperature; visualization using 3,3′-diaminobenzidine (Sigmafast DAB tablets – Sigma, Gillingham, United Kingdom) substrate solution; application of hematoxylin counterstain; dehydration in increasing concentrations of ethanol; clearing in xylene and mounting using DPX mounting medium (Sigma) and glass coverslips. Stained sections were viewed and photographed using a microscope and camera set (Leica Biosystems, Milton Keynes, United Kingdom). The same staining protocol was used for the additional United Kingdom patient cohort described in Supplementary Figure 2.

### Immunohistochemistry in the Prognostic Study

The protocol adopted in this part of the research was the routine technique used in the São Nicolau Laboratory (Cuiaba/Brazil), a pathology lab with extensive expertise in IHC. All branded solutions and buffers were purchased from Cell-Marque^TM^/Sigma-Aldrich (Rocklin, CA, United States). Four μm tissue sections were dewaxed in xylene and rehydrated as previously described. After a wash step in distilled water, slides were immersed in Trilogy^TM^ pre-treatment solution and incubated at 96°C for 30 min for epitope retrieval. After this, the slides were washed in phosphate buffered saline (PBS), Peroxide block^TM^ solution was added and samples were incubated for 20 min. After another PBS wash, the primary antibody solution (same concentration as those described above) was placed onto the samples and incubated for 20 min at room temperature. After washes in PBS, HiDef Detection^TM^ amplifier (secondary antibody solution) was applied to the slides for 15 min. After a further PBS wash, the former step was repeated using HiDef Detection^TM^ detector (a horseradish peroxidase polymer solution). Finally, color development was performed by incubating the slides with DAB substrate^TM^ chromogen. Stained slides were counterstained, dehydrated and mounted as described above. Slides were photographed using an Axio Scope.A1 microscope coupled with an AxioCamHRc camera (Zeiss, Oberkochen, Germany). Some samples were stained using both the protocol described here and that in the previous section and these confirmed that the staining patterns were similar (see Supplementary Figure 1).

### Immunohistochemistry Scoring System

Scoring was performed electronically using the software ImageJ (publicly available at rsbweb.nih.gov/ij/) (IMAGEJ). The images were initially edited in Image J to exclude non-epithelial/non-cancerous/stromal tissues. For cytoplasmic assessment, the plugin *IHC Profiler* was used (17). Based on the readings produced by this tool, we calculated a modified IHC Profiler score = [(% of negative) × 0] + [(% of low-positive) × 100] + [(% of positive) × 200] + [(% of high-positive) × 300], with final scores ranging from 0 to 300. For nuclear scoring, the plugin *ImmunoRatio* was used (18). It assesses the percentage of positive nuclei based on a threshold setting. Two microscopy fields (×400) containing at least 100 stained epithelial (in control cases) or cancer cells each were analyzed per sample.

### Statistical Analysis

Comparisons of continuous variables were carried out using Mann–Whitney *U* test or Kruskal–Wallis test followed by the Dunn-Bonferroni test for *post hoc* comparison. Categorical data were compared using the Chi-square test (or Fisher’s exact test in case of less than five expected counts per cell in the contingency table). For the survival analysis, groups were assessed using the Kaplan-Meier method, and survival curves were compared by log-rank tests. When significant differences were observed, Cox proportional hazards model was used for multivariate analysis. Two-sided *p*-values <0.05 were accepted as significant in the entire study. All statistical analyses were performed using the software IBM^®^ SPSS^®^ Statistics version 22 and R packages.

## Results

### Combination of Proteomic and Transcriptomic Datasets

Our previously published proteomic data (14) was integrated with the DNA microarray data from the double-mutant *Apc*- and *Myc*-deficient (*AhCre^+^Apc^fl/fl^Myc^fl/fl^*) mice as shown in Supplementary Table 1. Of the 93 up-regulated proteins from the iTRAQ analysis, only one also showed up-regulation of mRNA in the intestine of *AhCre^+^Apc^fl/fl^Myc^+/+^* mice and no change in the intestine of *AhCre^+^Apc^fl/fl^Myc^fl/fl^* mice using the criteria described in the *Materials and Methods* section. This protein was NAP1L1.

### Evaluation of *Nap1l1* mRNA Expression in Mouse Small Intestine

**FIGURE 1:**
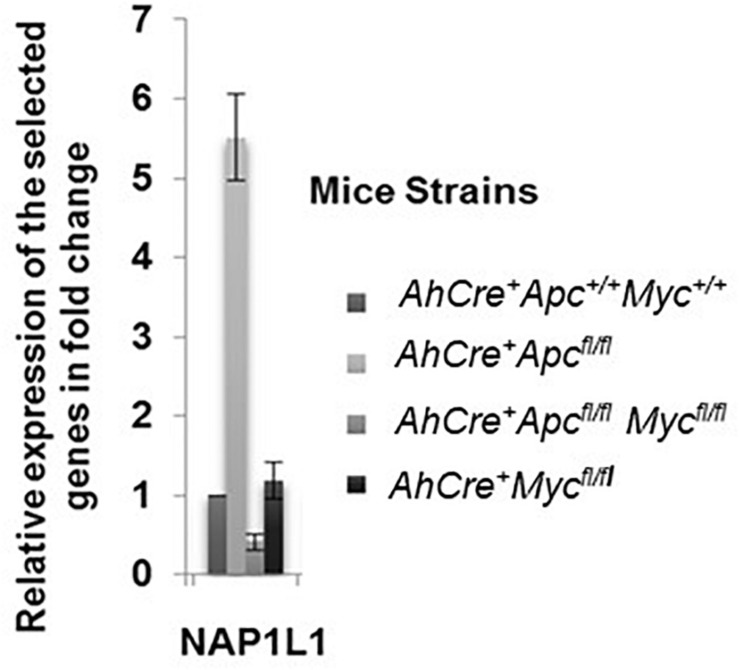
Relative expression of *Nap1l1* mRNA extracted from small intestinal epithelial samples from induced *AhCre^+^Apc^+/+^Myc^+/+^*, *AhCre^+^Apc^fl/fl^*, *AhCre^+^ Apc^fl/fl^ Myc^fl/fl^*, and *AhCre^+^Myc^fl/fl^* mouse models (four mice in each group) showing the fold change in mRNA expression for each gene relative to *AhCre^+^Apc^+/+^Myc^+/+^*. Error bars: standard error (SE). *p* < 0.05 for *AhCre^+^Apc^fl/fl^* compared to other groups.

In order to validate whether *Nap1l1* mRNA was upregulated following conditional *Apc* deletion in the mouse intestinal epithelium, qRT-PCR was carried out using mRNA extracted from small intestinal epithelial cell extracts from *AhCre^+^Apc^*fl/f*^Myc^fl/fl^*, *AhCre^+^Apc^fl/fl^*, and *AhCre^+^Myc^fl/fl^* mice, 4 days post induction. We compared relative mRNA expression in these transgenic mouse cohorts with the mRNA expression levels in *AhCre^+^Apc^+/+^Myc^+/+^* (wild-type) mice. Results are shown as fold change relative to the wild-type mice (Figure 1). This analysis confirmed that *Nap1l1* mRNA expression was significantly increased following *Apc* deletion in a *Myc*-dependent manner.

### Evaluation of *NAP1L1* mRNA Expression in Human Colorectal Cancer Samples

**FIGURE 2:**
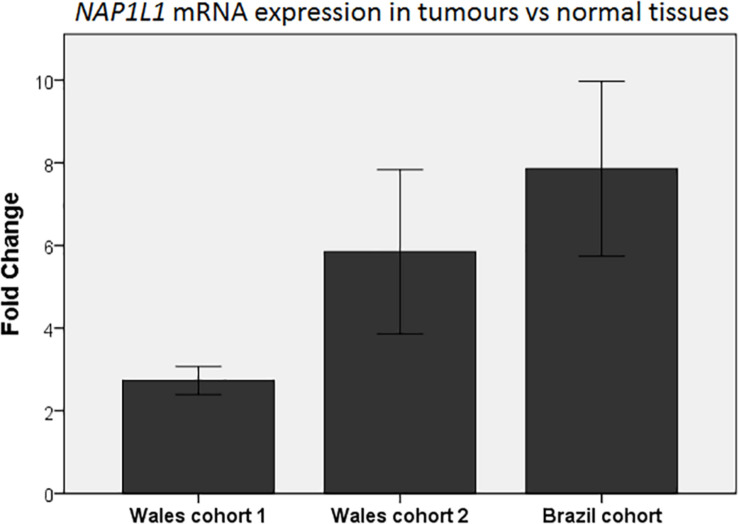
qPCR analysis of *NAP1L1* expression in CRC tumors presented as fold-change relative to normal tissues in different cohorts. Each column represents the relative quantification (fold-change) of *NAP1L1* mRNA expression in CRC compared to the respective normal control (normal control expression mean = 1). Wales cohort 1, mean fold-change = 2.7; *p* < 0.05 (18 paired colorectal cancer and adjacent normal colonic tissue samples). Wales cohort 2, mean fold-change = 5.8; *p* < 0.001 (30 paired colorectal cancer and adjacent normal colonic tissue samples). Brazil cohort, mean fold-change = 7.9; *p* < 0.001 (10 normal samples from healthy individuals without colorectal neoplasia and 25 colorectal cancer samples). Mann–Whitney *U* test. Error bars: ±1 SE.

*NAP1L1* was then analyzed in three cohorts of human CRC samples. We assessed the expression of *NAP1L1* firstly in mRNA from CRC tissues and matched normal mucosa from 18 patients supplied by the Wales Cancer Bank. *NAP1L1* demonstrated statistically significant elevated mRNA levels in CRC samples (Figure 2, Wales cohort 1). We next performed a confirmatory study using a different set of 30 samples from the same Tissue Bank, and we observed consistent results (Figure 2, Wales cohort 2). In order to further validate the findings, we repeated the experiment using a different platform (in terms of equipment and reagents) and compared a cohort of 10 normal colonic samples (individuals without any colonoscopic evidence of colorectal neoplasia) and 25 CRC samples from Brazil (Figure 2, Brazil cohort). Once more, significantly increased levels of *NAP1L1* mRNA were observed in CRC specimens and this time the comparison was with normal colonic tissue from patients who had no evidence of colorectal neoplasia.

### Confirmation of Differential Immuno-Expression of NAP1L1 in Human Colorectal Tissues

Immunohistochemistry for NAP1L1 was then performed in colorectal tissue samples from a different cohort of 32 patients, as described in section “Materials and Methods.” Cancer tissues and the matched unaffected mucosa collected 10 cm from the primary lesion were analyzed. Scoring was performed electronically using the software ImageJ (publicly available at rsbweb.nih.gov/ij/) and the plugins IHC Profiler for cytoplasmic scoring (resulting in a modified H-score ranging from 0 to 300)(17) and ImmunoRatio for nuclear scoring (ranging from 0 to 100%)(18). The samples were subdivided into two groups who had early stage (11 samples encompassing stages I and II) and late stage (21 samples including stages III and IV) CRC.

**FIGURE 3:**
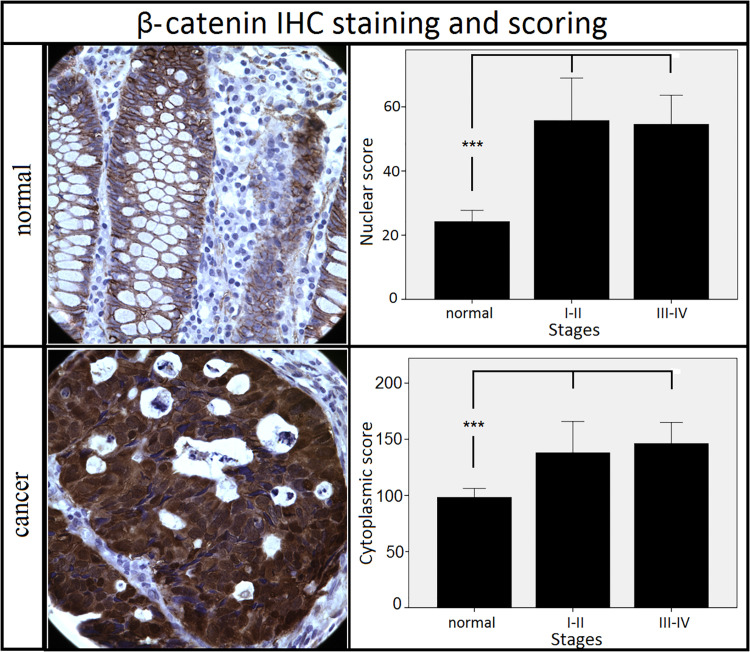
Staining patterns and scoring results for β-catenin. Nuclear and cytoplasmic staining of β-catenin increased in cancer tissues when compared to the adjacent mucosa. No difference was observed between different stages of cancer. ***p < 0.001 (Kruskal–Wallis test followed by *post hoc* Dunn-Bonferroni test for pair-wise comparisons). Error bars: ±2 SE. Sample numbers: normal = 32, stages I–II = 11, stages III–IV = 21. Magnification: 630×.

**FIGURE 4:**
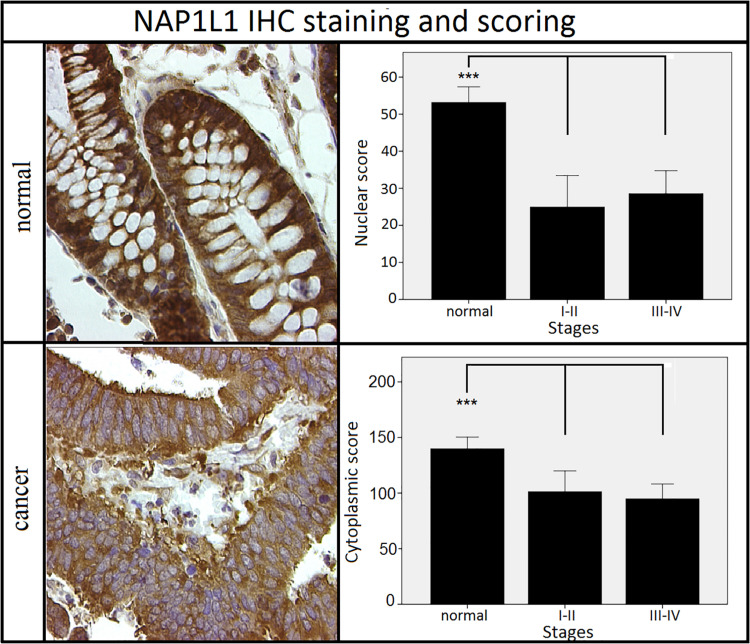
Staining patterns and scoring results for NAP1L1. NAP1L1 nuclear and cytoplasmic scores were decreased in the cancer groups when compared to normal adjacent tissues. No difference was observed between different stages of cancer. ***p < 0.001 (Kruskal–Wallis test followed by *post hoc* Dunn-Bonferroni test for pair-wise comparisons). Error bars: ±2 SE. Sample numbers: normal = 32, stages I–II = 11, stages III–IV = 21. Magnification: 630×.

We initially assessed the expression of β-catenin to confirm Wnt pathway activation and to validate the electronic scoring methods (Figure 3). A clear and statistically significant increase in both nuclear and cytoplasmic localization of β-catenin was observed in cancer tissues compared to the adjacent mucosa, as expected based on previous literature (19–21), thus validating our scoring system. Using the same scoring methods, we observed an opposite staining pattern for NAP1L1. A clear and statistically significant decrease in both the nuclear and cytoplasmic expression of NAP1L1 was seen in CRC tissues relative to the adjacent mucosa (Figure 4). No difference was detected between early and late stage tumor groups for both markers.

We also performed a confirmatory analysis using a different cohort of 19 patients from the Countess of Chester Hospital NHS Foundation Trust (Chester, United Kingdom). This analysis used a slightly different manual scoring method as described in Supplementary Figure 2. The findings were very similar to those demonstrated in the Brazilian patients and again demonstrated decreased NAP1L1 expression in the CRC samples (Supplementary Figure 2).

### NAP1L1 Nuclear Expression Is a Strong Predictor of Survival in Late Stage CRC

Having demonstrated decreased NAP1L1 immunohistochemical expression in CRC samples, we investigated whether the expression pattern had any effect on patient outcome. We analyzed a further cohort of 75 CRC cases diagnosed between 2004 and 2012. Median follow-up was 84.7 months (range 48–153 months). Given the relatively small number of cases, cancer stages were again combined into two groups: early stage (stages I and II) and late-stage (stages III and IV). Immunohistochemistry was conducted as described in section “Materials and Methods.” Table 1 describes the characteristics of the patients included in this analysis.

**TABLE 1:** Characteristics of the patients included in the prognostic analysis.

Characteristics	Patients (*n* = 75)
**Mean age** (range)	59.7(33−84)
**Gender**	
Male	43(57.3%)
Female	32(42.7%)
**Stage**	
I–II	28(37.3%)
III–IV	47(62.7%)
**Grade**	
Well differentiated	23(30.6%)
Moderately differentiated	50(66.6%)
Poorly differentiated	2(2.6%)

Initially, using mortality status as the binary event of interest, ROC curves were generated. The area under the curve (AUC) was 0.58 for the nuclear score and 0.60 for the cytoplasmic score. Cut-offs were determined either by manually assessing the best balance between sensitivity and specificity or electronically by the use of the software Cutoff Finder (22) and X-Tile (23). All methods suggested the same cut-off for nuclear staining: 32% (of positive nuclei). Use of this threshold resulted in a sensitivity of 61% and a specificity of 67.5% for discriminating mortality status. For cytoplasmic staining, sensitivity/specificity optimization suggested a cut-off of 135 (in a range from 0 to 300), yielding a sensitivity of 57% and a specificity of 54%. The electronic tools suggested higher cut-offs (167 and 168). Although these resulted in a higher specificity (92%), the sensitivity was low (29%). Despite these differences, the prognostic results were similar, so the cytoplasmic cut-off of 135 was selected to describe the results.

The prognostic cohort was therefore divided into two groups with low-expression and high-expression of NAP1L1. Groups were similar in terms of age, gender, stage and grade. Table 2 shows the clinicopathological characteristics of the groups according to the nuclear expression of NAP1L1. Similar balanced distribution was also observed for cytoplasmic expression.

**TABLE 2:** Clinicopathological characteristics according to NAP1L1 nuclear expression.

Characteristics	Low nuclear expression (*n* = 34)	High nuclear expression (*n* = 41)	Two-sided *p*-values
**Mean age**	61.9	57.8	0.157
**Gender**			0.648
Male	17	24	
Female	16	18	
**Stage**			0.338
I–II	10	18	
III–IV	23	24	
**Grade**	.	14	0.351
Well differentiated	9	14	
Mod differentiated	24	26	
Poorly differentiated	0	2	

*No significant difference between groups was observed. Mean age was compared by *t*-test. Categorical variables were compared by Chi-square test or Fisher’s exact test.*

**FIGURE 5:**
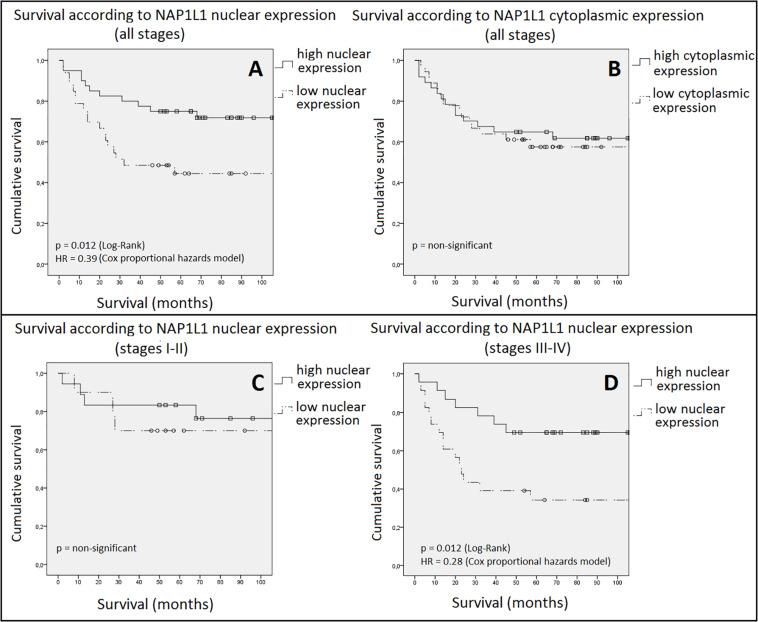
Cumulative survival according to NAP1L1 expression. In panel **(A)**, nuclear staining is assessed. A highly significant (*p* = 0.012) and clinically relevant (HR = 0.39 [95%CI: 0.17–0.87]) difference in survival between groups was observed favoring the high expression group. In panel **(B)**, cytoplasmic staining, no significant difference was observed. In panel **(C)**, nuclear expression for early stage disease (stages I and II) is assessed. No difference in survival was observed. Panel **(D)** shows the results for late stage disease (stages III and IV). A statistically significant difference was observed favoring the high nuclear expression group (HR: 0.28 [95%CI: 0.11–0.71]). Squares and circles = censored cases. HR: hazard ratio. CI: confidence interval.

Using the Kaplan–Meier method, cumulative survivals for the two groups (high and low nuclear expression) were compared. Initially, groups were assessed as a whole, regardless of disease stage (Figure 5A). A clear difference in cumulative survival was observed according to nuclear NAP1L1 staining (*p* = 0.012, log-rank test). In the multivariate analysis including age, gender, stage and grade (Cox proportional hazards model), the nuclear score was independently associated with cumulative survival. The high nuclear expression group exhibited a hazard ratio (HR): 0.39 ([95%CI: 0.17–0.87]; *p* = 0.02), denoting a 61% reduction in cumulative mortality in this group. As a result, the estimated 5-year survival was 44.4% in the low expression group and 75% in the high expression group. Median duration of survival was 32 months in the low expression group, whilst it was not reached for the high expression cohort. The only additional variable also associated with survival was tumor stage (HR: 2.55 [95%CI: 1.01–6.43]; *p* = 0.047), an expected finding since stage is a known prognostic factor in CRC. These results strongly suggest an association between NAP1L1 nuclear staining and survival in CRC patients. Conversely, cytoplasmic NAP1L1 staining was not associated with survival (Figure 5B) or with any other clinicopathological variable.

We then analyzed survival according to NAP1L1 nuclear expression in different stage groups (Figures 5C,D). For early stage disease, no significant difference in survival was found. By contrast, a highly significant difference in survival was observed for the cohort containing stages III and IV tumors. Multivariate analysis once again demonstrated that NAP1L1 nuclear score was an independent prognostic factor in CRC patients. The calculated HR (0.28 [95%CI: 0.11–0.71]; *p* = 0.008) was even more notable than that observed for the entire cohort, now suggesting a 72% reduction in cumulative mortality. The 5-year survival advantage for high expression tumors was also greater: 70%, versus 34% for low expression cancers. Median survival was only 23 months in the low expression group and, again, was not reached in the high expression cohort.

## Discussion

The discovery of novel CRC biomarkers to assist in early diagnosis, prognostic stratification and prediction of response to treatment remains an unmet medical need. We hypothesized that the study of animal models of CRC based on transgenic *Apc* gene inactivation could lead to the discovery of novel useful CRC biomarkers in humans.

By combining transcriptomic and proteomic analyses of small intestinal tissue from transgenic mice in which *Apc* and/or *Myc* had been specifically deleted, we identified NAP1L1 as the only gene/protein that showed significantly altered expression in *Apc* and *Myc*-dependent manners in all analyses. We confirmed these findings using qPCR in mouse small intestine and additionally demonstrated that *NAP1L1* mRNA expression was increased in human CRC. It was unfortunately not possible to study whether there was any altered NAP1L1 expression in the colon of the *AhCre* mouse model as there is no Cre mediated recombination in the colon of these mice following injection of β-naphthoflavone and they have no colonic phenotype (24).

NAP1L1 is a highly conserved histone chaperone protein which is one of five NAP1-like proteins in mammals (21, 22). It has been suggested to play a role in mediating nucleosome formation and regulation of the H2A-H2B complex as well as nucleosome assembly (25), cell cycle progression, and cell proliferation (26). It has also been linked to embryogenesis and tissue differentiation (23–25). Few researchers have previously studied *NAP1L1* expression in cancer cell lines or tissues. Drozdov et al. compared small intestinal neuroendocrine tumors (NETs) and normal enterochromaffin cell preparations, and showed a 13.7-fold increase in *NAP1L1* expression in tumor tissues (27). However, no analysis of the adjacent mucosa was performed. Kidd et al. also suggested that *NAP1L1* was increased in NETs but not in CRCs (28). Line et al. evaluated *NAP1L1* mRNA expression in CRC and adjacent tissues as a secondary endpoint in a study primarily aimed at finding sero-reactive biomarkers (29). They showed that, among 15 cases of CRC, seven exhibited moderate increases in *NAP1L1* expression (ranging from 2.9 to 9.3-fold) and eight cases showed expression levels similar to those in the corresponding adjacent mucosa. A recent paper has also demonstrated that NAP1L1 is a prognostic biomarker and contributes to doxorubicin chemotherapy resistance in hepatocellular carcinoma (30).

Immunohistochemistry is used in routine clinical practice to assess the expression of proteins with prognostic or predictive value in other types of cancer such as breast (31) and lung carcinomas (32), soft tissue sarcomas (33), and lymphomas (34). Given the absence of a standard scoring method for NAP1L1, we initially decided to assess both the nuclear and the cytoplasmic expression of the protein in our samples using electronic tools. Our results showed that NAP1L1 expression was decreased both in the nucleus and the cytoplasm of CRC tissues when compared to the normal adjacent mucosa.

This was a somewhat unexpected finding, given the increased expression of *NAP1L1* mRNA in animal models and in human tissues. Such discrepancy between mRNA and protein expression has however previously been demonstrated for other cancer markers (35–37). Several processes could be responsible, such as posttranscriptional modifications, protein degradation, secretion via exocytosis or alterations in subcellular protein localization. For example a recent paper has reported that NAP1L1 undergoes alternative cleavage and polyadenylation in the more advanced stages of CRC (38). The full length isoform of NAP1L1 was overrepresented in the cytoplasmic fraction of a CRC cell line which had a more metastatic phenotype. This may therefore represent one mechanism to explain the altered NAP1L1 subcellular localization that is reported in CRC specimens in our current manuscript. Counterintuitively, our finding of increased gene expression in the initial screen may have been a response to reduced protein content and not the primary event. Further research is required in order to clarify this issue. Qiao et al. (39) demonstrated that knock down of *NAP1L1* increased cellular proliferation, disrupted normal cell development and distribution, and caused global deregulation of gene expression. These are classical hallmarks of cancer and of activated Wnt signaling. Qiao et al. also demonstrated that *Nap1l1* knockdown resulted in reduced RassF10 expression, low expression of which has been associated with poor survival in CRC patients in another study (40). Thus reduced NAP1L1 protein expression could be mechanistically associated with tumor progression.

We also assessed whether NAP1L1 expression was associated with prognosis. We retrospectively retrieved blocks from a cohort of CRC patients with more than 4 years follow up. Cut-offs for nuclear and cytoplasmic expression of NAP1L1 were established and a survival analysis was performed. Nuclear expression of NAP1L1 correlated with overall survival in CRC. High nuclear expression was independently associated with an increase in median survival and 5-year survival estimates. Subgroup analysis however showed that the survival correlation was limited to late stage tumors (stages III and IV). No association between NAP1L1 nuclear expression and clinicopathologic variables (age, gender, stage and grade) was observed. These findings suggest that the expression of NAP1L1 could potentially help in discriminating low and high-risk disease in stage III CRC cases and, also, to determine the aggressiveness of the disease in stage IV cancers. In both cases, this information could help to better define the best treatment approach.

We acknowledge some limitations in this study. Although positive and relevant findings were observed, the use of small clinical sample sizes may have limited our observations. This also meant that it was necessary to evaluate cancer stages as combined groups rather than individually. Better prognostic stratification in stage II disease is urgently needed to improve the treatment decision-making process and our data did not permit this. Moreover, analyzing larger cohorts of stage III and stage IV diseases separately would also be desirable, as these stages are associated with markedly different clinical outcomes.

This study provides proof of concept that the analysis of animal models of Wnt pathway activation may yield potentially useful CRC biomarkers in humans. We undertook a comprehensive assessment of NAP1L1 expression in animal models and clinical samples and our findings suggest that it could be a prognostic biomarker for CRC. Confirmatory research studying larger sample cohorts and a better assessment of the role of this protein in CRC carcinogenesis is now recommended before this marker can be introduced routinely into clinical practice.

## Data Availability Statement

The datasets generated for this study can be found in the Broad Institute Gene Set Enrichment Analysis (GSEA) M1755, M1756, M1757, and M1578.

## Ethics Statement

The studies involving human participants were reviewed and approved by the respective institutions, Brazilian National Commission for Research Ethics (CONEP); Wales Cancer Bank and Countess of Chester Hospital NHS Foundation Trust. The patients/participants provided their written informed consent to participate in this study. The animal study was reviewed and approved by the Cardiff University Animal Welfare Ethical Review Panel and UK Home Office.

## Author Contributions

JJ, DP, KR, and AC designed the research project. JJ, FM, and DP supervised CQ’s Ph.D. studies. JJ and DV supervised NA-K’s MRes studies. KR managed the mouse inter-crosses and collection of murine samples. FS performed the qPCR of human samples. CQ and NA-K performed the human sample collection, qPCR, immunohistochemistry staining, and scoring and data analysis. CQ, DP, and JJ drafted the manuscript and all other authors critically reviewed the manuscript and approved the final version submitted. All authors contributed to the article and approved the submitted version.

## Conflict of Interest

The authors declare that the research was conducted in the absence of any commercial or financial relationships that could be construed as a potential conflict of interest.
