# Supplementary material for: NAP1L1: A Novel Human Colorectal Cancer Biomarker Derived From Animal Models of Apc Inactivation
Source: Front Oncol. 2020 Aug 11;10:1565. doi: 10.3389/fonc.2020.01565 (PMC7431561; doi:10.3389/fonc.2020.01565)
Supplement: Supplementary file 2 [file Data_Sheet_1.docx]

Supplementary Material

**Supplementary tables and** **figure**

**Supplementary table 1^*^:** Comparison of proteomic and Affymetrix microarray data for small intestinal epithelial tissues from *AhCre^+^Apc^+/+^Myc^+/+^, AhCre^+^Apc^fl/fl^, AhCre^+^Apc^fl/f^Myc^fl/fll^* and *AhCre^+^Myc^fl/fl^* mouse models. Highlighted boxes show the proteins that met the criteria outlined in the Materials and Methods section, namely:

1) APC:WT >2 with P<0.05

2) APCMYC:WT = 0.75 to 1.25

3) APCMYC:APC <0.5 with P<0.05

*See Excel spreadsheet as a separate file (Supplementary table 1).

**Supplementary table 2: qRT-PCR primers used in animal experiments (mouse) and in the UK cohorts (human).**

| **Gene name** | **Species** | **Probe number** | **FWD seq** | **REV seq** |
| --- | --- | --- | --- | --- |
| ***ACTB*** | Human | #64 | **ccaaccgcgagaagatga** | **ccagaggcgtacagggatag** |
| ***Actb*** | Mouse | #64 | **ctaaggccaaccgtgaaaag** | **accagaggcatacagggaca** |
| ***NAP1L1*** | Human | #35 | **caagatttggatgatgttgaaga** | **aacagttagctgacgtgctttg** |
| ***Nap1l1*** | Mouse | #93 | **cggtcagagccagatgattc** | **tggttttcaaagtaacattctttcc** |

**Supplementary table 3: qRT-PCR assays used in the Brazil cohort.**

| **Gene Symbol** | - **Entrez Gene ID** | **Description** | - **TaqMan® assay Id^(*)^** |
| --- | --- | --- | --- |
| *NAP1L1* | 4673 | nucleosome assembly protein 1-like 1 | Hs00748775_s1 |
| *ACTB* | 60 | actin, beta | Hs99999903_m1 |

###
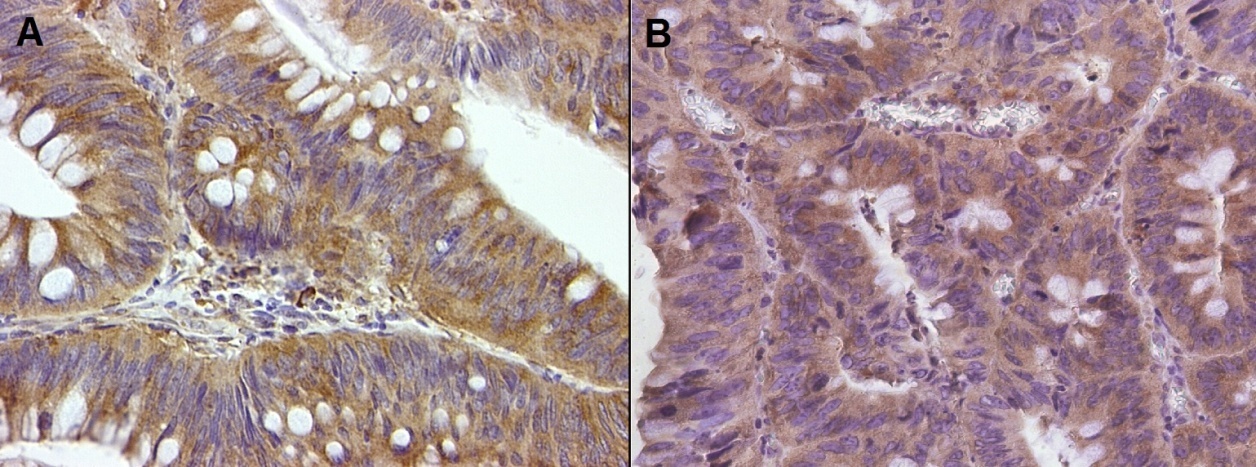


**Supplementary figure 1.** Comparison of NAP1L1 staining patterns observed with the IHC protocols used in the initial validation study (A) and in the prognostic study (B), using the same sample. Similar staining intensity and localisation are observed. Differences in colour shade and background are due to the use of different cameras for recording the images. Magnification: 600x.


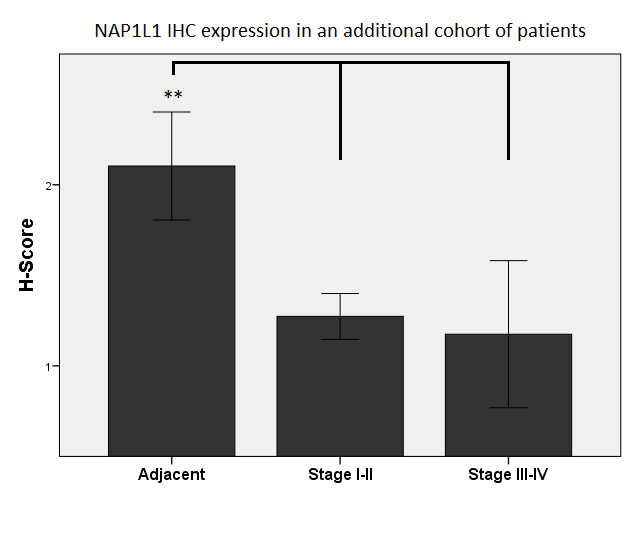


**Supplementary figure 2**. IHC expression of NAP1L1 assessed manually using a modified H-score. This assessment was performed in a different cohort of patients, using a different scoring method. Samples were obtained from the Countess of Chester Hospital NHS Foundation Trust, Chester, UK. Tissues were scored according to the level of staining observed in the nucleus relative to the cytoplasm. Score 0 was represented by low level staining of both the nucleus and cytoplasm, score 1 by increased staining of the cytoplasm, but little or no staining of the nucleus, score 2 by equal staining of both cytoplasm and nucleus, and score 3 by nuclear staining which was darker than that present in the cytoplasm. The “Cell Counter” plugin available on imageJ [rsbweb.nih.gov/ij/] was used to score all visible cells on 40x images. A final H-score was then calculated using the equation (%Score0x0) + (%Score1x1) + (%Score2x2) + (%Score3x3). Results showed a decreased H-score in cancer tissues when compared with the adjacent uninvolved mucosa, supporting the main findings described in this study. However, this was a relatively small cohort of samples (7 adjacent normal samples, 8 stage I-II colorectal cancers and 11 stage III-IV colorectal cancers; **Kruskal-Wallis test p< 0.01). In view of the smaller sample size and different scoring protocol employed, these results have been provided as a supplement to the main manuscript. Error bars: ± 2 SE.
